# Sealing faults and fluid-induced seismicity

**DOI:** 10.1098/rsta.2023.0418

**Published:** 2024-08-09

**Authors:** Zahra Esmaeilzadeh, David W. Eaton, Navid Hosseini, Danial Zeinabady

**Affiliations:** ^1^ Department of Earth, Energy & Environment, University of Calgary, Calgary, Alberta, Canada; ^2^ Department of Earth Science and Engineering, Imperial College London, London, UK; ^3^ Shear Frac Group, LLC, Calgary, Alberta, Canada

**Keywords:** induced seismicity, sealing fault, hydraulic fracturing, geological storage

## Abstract

Sealing faults are nearly impermeable barriers that can form boundaries between subsurface pore-pressure domains. In hydrocarbon systems, sealing faults commonly form part of a structural trap; they are thus important elements for future storage of CO_2_ and other gases in depleted reservoirs. The Triassic Montney Formation in western Canada hosts low-permeability gas reservoirs containing sealing faults that have previously been assumed to compartmentalize pressure domains. In this study, we show that the distribution of induced seismicity associated with hydraulic fracturing (HF) exhibits a statistically significant spatial correlation with zones of high lateral gradient in pore pressure. These high-gradient zones are interpreted as sealing fault systems. The largest induced seismicity sequence, including a 4.5 M_L_ mainshock on 30 November 2018, occurred during HF treatments in two horizontal wells, between which there is an exceptionally large contrast (~10 MPa) in measured pore pressure. Numerical simulation of a simplified model of a hydraulic fracture intersecting a nearby vertical fault, followed by fault rupture using rate-and-state friction rheology, generates results that are in good agreement with observed strike-slip faulting near one of the HF wells. Our study demonstrates that sealing faults exhibit previously unrecognized behaviour that may be important for understanding induced seismicity risk.

This article is part of the theme issue ‘Induced seismicity in coupled subsurface systems’.

## 1. Introduction

Faults can act as permeable conduits for fluid flow in the subsurface [1–3], but they can also serve as nearly impermeable barriers, known as sealing faults [2,4,5]. In petroleum systems, fault seals are widely recognized as a key component of trapping mechanisms, with fault sealing attributed to the juxtaposition of lithologies with contrasting permeability or to an impermeable fault core [6,7]. Fault sealing conditions can be created during diagenesis by mineral precipitation or various creep mechanisms [8], hydration or dehydration reactions [9] or by generation of ultra-fine gouge materials due to fault wear [10]. Fault permeability is also important for understanding leakage in subsurface gas storage systems, including geological carbon storage [11], where changes in permeability due to mineralization have been documented [12]. The presence of a sealing fault is difficult to determine remotely using seismic imaging, often requiring indirect inference based on measured changes in pore pressure, i.e. the fluid pressure within pore space in a porous medium, across a fault [7].

Fluid-induced seismicity can be triggered by an increase in pore pressure on a fault (e.g. [13–15]) or by changes in the poroelastic stress state (e.g. [16,17]. The physical mechanisms are generally well understood, as an increase in pore pressure will lead to a reduction in effective normal stress and thus a reduction in frictional fault strength [13,18,19]. Similarly, poroelastic models show that fault destabilization can occur due to changes in stress conditions without the direct pore-pressure effect [16,20]. In sedimentary basins, *in situ* pore pressure can exhibit significant spatial variability due to the effects of differential subsidence, uplift, hydrocarbon generation and fluid migration in the presence of permeable pathways and barriers—leading to varying degrees of local susceptibility to fault activation [21]. In addition to spatial variability, numerous studies show that fault permeability evolves throughout the earthquake cycle, including significant reduction in permeability during interseismic hold periods [22–25].

Chang & Segall [16] carried out numerical simulations for a layered poroelastic medium that considered seismicity rate based on a modified rate-and-state friction model. Several of their models incorporated sealing faults, characterized by large initial across-fault gradients in stress. Their numerical analysis showed that elevated pore pressure in the surrounding rock mass can contribute to poroelastic stresses that can, in some cases, promote slip [16]. Using a similar numerical simulation approach, Chang *et al*. [26] showed that lower permeability faults can exhibit higher seismicity rates after shut-in than more permeable faults, due to poroelastic stressing and delayed diffusion. For models that incorporated a sealing fault, Chang *et al*. [26] found that direct diffusion into the fault is inhibited but pore-pressure perturbations are nevertheless observed due to poroelastic effects; however, injection-driven expansion of the host reservoir can generate compression within the fault zone that acts to stabilize the fault.

The aim of this study is to investigate relationships between sealing faults, subsurface pore-pressure domains and fluid-induced seismicity. The first part of this article adopts an empirical approach based on a case study in northeastern British Colombia, Canada. In this region of the Western Canada Sedimentary Basin, both fluid-induced seismicity (mainly from hydraulic fracturing) and pore-pressure domains are well characterized, allowing robust statistical inferences to be made. Here, the Triassic Montney Formation hosts low-permeability gas reservoirs containing sealing faults, which have previously been conjectured to compartmentalize pressure domains [27] within a regional transition from overpressured to normally (hydrostatically) pressured parts of the basin. The largest induced earthquake in this area, a 4.5 M_L_ event on 30 November 2018 [28,29], took place during hydraulic fracturing (HF) of two horizontal wells, between which an exceptional contrast in pore pressure (~10 MPa) occurs over a distance of 1.2 km [30,31]. The 2018-induced seismicity sequence activated a strike-slip fault proximal to one of the HF wells. The second part of this article discusses a series of numerical simulations using these attributes. The models consider steady-state pore-pressure conditions in the presence of a sealing fault that separates high- and low-pressure domains within the Montney Formation. A HF simulation is used to estimate the pore-pressure distribution on the fault in the nucleation region of a small earthquake at the intersection of the HF with the fault. The calculated pore-pressure conditions are then used in a rupture model that considers rate-and-state frictional rheology of the fault to simulate hybrid (fast + slow) slip on the fault. This set of simulations yields results that are generally consistent with field observations.

## 2. Case study

The case study focuses on the Lower Triassic Montney Formation, a westward-prograding subsurface wedge of siliciclastic rocks (primarily siltstone) with a maximum thickness of 340 m [32] deposited in various settings ranging from deep-water to upper-shoreface estuarine environments [33]. The Montney Formation hosts a variably overpressured unconventional (low permeability) hydrocarbon fairway [34]. Overpressure is a pore-pressure regime in which *in situ* pore pressure exceeds the hydrostatic gradient (approx. 10 kPa/m). Mechanisms for creating overpressure in low permeability (tight) reservoirs included hydrocarbon generation and inhibited pore fluid drainage from the pores including sealing faults. During uplift and exhumation, the Montney tight hydrocarbon system underwent significant de-pressurization due to the leakage of fluids, primarily gas [35]. This process left localized regions of overpressure as vestiges of the initially overpressured regime before uplift.

### (a) Methods

This study uses pore-pressure data for the Montney Formation mainly compiled by Enlighten Geoscience [27], who performed a comprehensive study on pressure and stress mapping and fault-slip potential analysis in the Kiskatinaw Seismic Monitoring and Mitigation Area (KSMMA). The KSMMA was established by the BC Energy Regulator to implement special requirements for induced seismicity monitoring [36]. Different data sources were used by Enlighten Geoscience [27] to estimate pore pressure within the low-permeability Montney Formation. In this environment, pore-pressure data are usually extracted from diagnostic fracture injection tests (DFITs) and routine pressure survey tests (PST). DFITs, also known as minifrac tests, include a short injection test followed by a few hours of fall-off. The post-shut-in pressure decay is used to estimate reservoir parameters needed for fracture design, including reservoir pore pressure, fracture pressure, fracture closure pressure (approximately equivalent to minimum horizontal stress) and permeability. The PST data include tests from the following categories: bottom hole build-up, bottom hole static gradient, acoustic well sounder and minifrac tests. Additional Montney bottom-hole pressure measurements have been added to the previous compilation [30] and are also included here. Pore-pressure measurements exhibiting extreme sub-hydrostatic gradient less than 6 kPa/m (the lowest limit in the DFIT data) are assumed to be erroneous and are excluded from further analysis.

**Figure 1.**
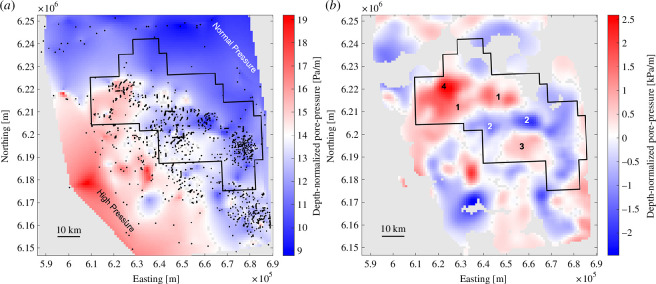
(*a*) Map of depth-normalized pore-pressure for the Montney Formation in northeastern British Colombia, Canada, based on kriging of measured values (black dots). The black polygonal outline shows the Kiskatinaw Seismic Monitoring and Mitigation Area (KSMMA), which has special requirements for induced seismicity monitoring [36]. An overall pore-pressure transition is evident from high (over-) pressure in the deep-basin region in the southwest to normal pressure in the northeast. (*b*) Residual pore-pressure map obtained from gridded data in (*a*) by subtracting a best-fitting quadratic surface and smoothing using a 5 km low-pass filter. Masked-off grey areas show grid locations where kriging yielded a null value and/or the distance to the nearest measurement location exceeds 5 km. Labelled features: 1 = septimus high, 2 = saturn low, 3 = doe high, 4 = monias high. UTM map projection (zone 10).

Figure 1*a* shows pore pressure in the Montney Formation from the compiled dataset, normalized by depth. Starting with the initial irregular spatial sampling, kriging was used to generate a regular grid of values at 1 km spacing. We used Gaussian process regression in MATLAB to carry out this step, which generates non-parametric, kernel-based probabilistic models based on user-selected parameters. We chose the rational quadratic kernel with a separate length scale per predictor as the form of the covariance function as this minimized the misfit, resulting in standard deviation of 1.33 kPa/m. Grey regions of the map show grid points that yield null values when kriging is applied. The map illustrates a clear regional trend in pore pressure of the Montney Formation, from overpressured in the southwest, within the deep basin near the Rocky Mountain deformation front, to normally pressured in the northeast. Lobate secondary features are superimposed onto this trend. Although our compilation contains some additional data points, this map is very similar to pore-pressure mapping presented by Enlighten Geoscience [27], who interpreted pore pressure in this region to be compartmentalized into distinct domains of high and low pore pressure. Enlighten Geoscience [27] proposed that boundaries for some of the pore-pressure domains coincide with locations of mapped faults. However, these faults are mainly derived from unpublished sources, and inconsistencies in published fault maps have been noted [37]. To avoid this source of uncertainty we use a different approach. We obtained a residual pore-pressure map by removing the estimated regional trend and computing the lateral gradient of depth-normalized pore pressure because (as discussed further below) high lateral gradient in pore pressure provides a proxy for sealing fault systems.

Figure 1*b* shows a map of residual pore pressure for the Montney Formation, constructed by regional-residual separation followed by spatial smoothing. The regional field was approximated using the best-fitting quadratic surface obtained with the MATLAB function *psfit*. After subtracting this surface, we applied a simple low-pass smoothing filter using two-dimensional convolution of the gridded data with a boxcar function. After carrying out a sensitivity analysis for the size of the filter mask (see the electronic supplementary material), we selected a 5 × 5 km filter mask as this was judged optimal with respect to trade-offs between desirable and excessive smoothing of the data given the irregular distribution of sample points. After applying the smoothing filter, we removed grid points whose nearest measured value is greater than 5 km. Although there are significant uncertainties with our approach, the resulting map (figure 1*b*) reveals several arcuate trends and/or localized features of high and low anomalies, many of which coincide with features identified by Enlighten Geoscience [27]. For example, the saturn low is a curvilinear low pore-pressure domain with a general east-west trend. The septimus high and doe high flank the saturn low to the north and south, respectively. Near the northwestern corner of the KSMMA, the Monias High contains the highest residual pore-pressure values.

**Figure 2.**
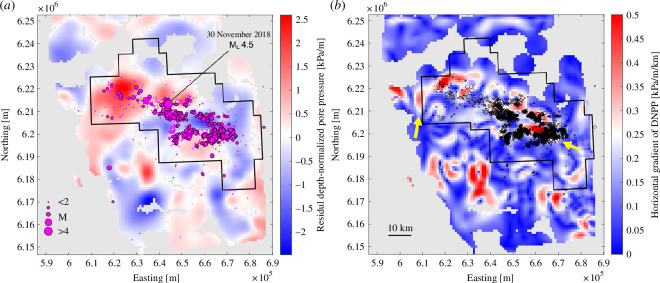
(*a*) Depth-normalized pore-pressure map from figure 1*b*, with inferred hydraulic-fracturing (HF) induced seismicity overlaid (magenta symbols). The epicentral distribution appears to cross-cut high-pressure and low-pressure domains. This distribution reflects locations of HF wells in addition to fault systems that are prone to reactivation [38]. The black polygonal outline shows the KSMMA, which has special requirements for induced seismicity monitoring [36]. (*b*) Horizontal gradient of depth-normalized pore pressure. HF-induced seismicity is overlaid with open black symbols, using the same magnitude scaling as in (*a*). Arrows highlight several inferred fault systems within the Fort St. John Graben structural corridor [37]. UTM map projection (zone 10).

Figure 2 highlights the spatial relationship of (inferred) HF-induced seismicity and pore-pressure domains. The seismicity data were downloaded from the BC Energy Regulator website (see Data Accessibility) for the period 6 March 2000 to 31 March 2022. To remove events that are probably unrelated to HF operations, 6939 seismic events were filtered based on two different sets of conditions. The first set of conditions considered earthquakes that occurred after the start of HF operations, prior to 90 days after the completion of operations, and within 7 km of HF wells. Applying these conditions reduced the catalogue to 5778 earthquakes. A second set of conditions was more restrictive by considering only events within 5 km of HF wells and 30 days of the last HF operations. This resulted in 5541 events, nearly the same as the previous subset. Since the variation in outcomes between these filtering scenarios was relatively minor, we use the 7 km and 90 day criterion to ensure that all seismic events that are potentially associated with HF activities have been included. In either case, the distribution of seismicity is strongly clustered in parts of the central KSMMA. The clusters of seismicity are generally elongated, delineating approximately east–west trends. Two prominent bands of (induced) seismicity roughly flank the Saturn Low to the north and south. However, contrary to larger-scale findings of Eaton & Schultz [21], who postulated that induced seismicity occurs preferentially in areas of elevated pore pressure, the seismicity clusters overlap both high-pressure and low-pressure domains.

Figure 2*b* shows a horizontal gradient map computed from the depth-normalized pore-pressure grid. Within the KSMMA, this reveals several features that correlate with structural corridors identified by Wozniakowska *et al*. [37] using formation tops. One feature, marked by an arrow on the east side of the map, shows a series of elongated features that align with the *en echelon* southern boundary of the Fort St. John Graben. Another feature, marked by an arrow on the west side of the map, marks the Monias structural corridor [37], a set of horst and graben features. The apparent correlation of these high-gradient zones with structural corridors suggests that there may be a spatial relationship with fault zones, consistent with previous interpretations in this area [27].

### (b) Results

**Figure 3.**
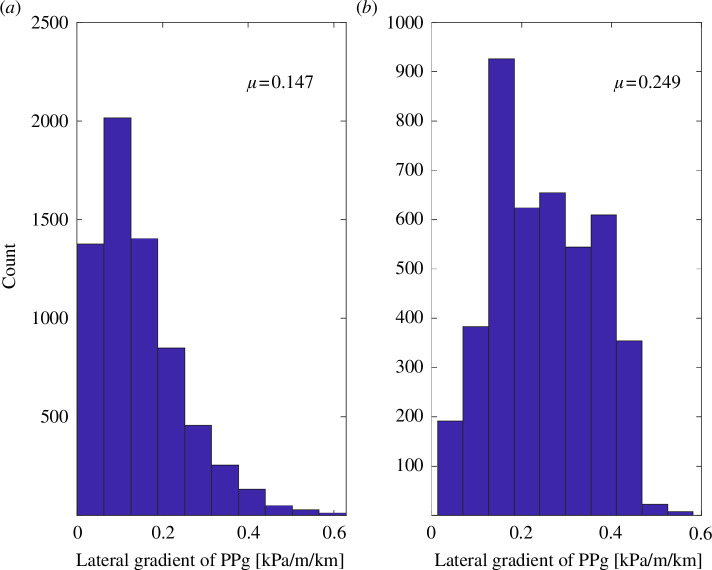
(*a*) Histogram of un-masked gradient values from figure 2*b*. The mean value (*μ*) of the distribution is 0.147 kPa/m/km. (*b*) Histogram of gradient values at epicentral locations from figure 2*a*. The mean value (*μ*) of the distribution (0.249 kPa/m/km) is significantly higher than in (*a*).

The seismicity clusters in figure 2*a* appear to align with elongated high-gradient anomalies in figure 2*b*. To evaluate this apparent alignment, we extracted the interpolated gradient value at every epicentre location for the seismic events that could be associated with HF operations. Figure 3 shows a histogram of these interpolated gradient values alongside a histogram of all gradient values mapped in figure 2*b*. The distribution of gradient values extracted at seismic event locations has a mean value of 0.249 kPa/m/km, compared with a mean value of 0.147 kPa/m/km for the entire map region. Bootstrap analysis was carried out using HF well locations, regardless of whether the wells had associated seismicity, as even within the KSMMA, the majority of HF wells have no associated induced seismicity. The HF wells were selected at random to generate a list of locations equal in length to the HF-associated subset of the seismicity catalogue. The pore-pressure gradient was then extracted at every selected location, and then the mean value of the distribution was determined. After 10 000 iterations, the mean gradient from all of these distributions was 0.174 kPa/m/km, with a standard deviation of 0.0016. This indicates that the well locations are slightly biased toward high-gradient regions, but not nearly as much as the seismicity. We, therefore, conclude that HF-associated induced seismicity is preferentially concentrated in areas of large horizontal gradients in pore pressure. As elaborated below, sealing fault zones are expected to be marked by anomalously high horizontal gradients in pore pressure.

**Figure 4.**
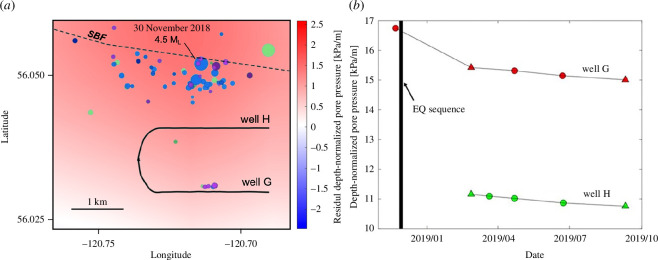
(*a*) Locations of seismic events associated with the 30 November 2018 induced seismicity sequence (modified from [29]). Blue, magenta and green symbols are scaled by magnitude and denote primarily reverse, strike-slip and normal faulting mechanisms, respectively. The sequence occurred mainly below the Montney Formation during hydraulic fracturing of wells G and H, which were drilled horizontally into the lower Montney Formation. SBF: the southern bounding fault of the Fort St. John Graben where it intersects the Carboniferous Debolt Formation. The SBF lies within a region of relatively high pore pressure, as shown by the background image of residual depth-normalized pore pressure (figure 2*a*). The background images are plotted with the same colour scale as in figure 2*a* for ease of comparison. A three-dimensional plot showing focal depth of these events is presented in the electronic supplementary material. A previously unmapped strike-slip fault (magenta symbols) is indicated by the distribution of small events near well G. (*b*) Temporal evolution of depth-normalized pore pressure in wells G and H, showing a nearly consistent difference of about 4.5 kPa/m between wells G and H. We attribute this difference to the presence of a sealing fault between the two wells. Circles show static gradient measurements and triangles show pressure-gauge build-up measurements (see the electronic supplementary material).

Next, we consider, in closer detail, the 30 November 2018 induced seismicity sequence. The location of this sequence within the KSMMA is marked in figure 2*a*. During this sequence, a 4.5 M_L_ mainshock was followed by two aftershocks within an hour, with magnitudes of 4.3 M_L_ and 3.6 M_L_ [28,29,39]. At the time that this sequence was initiated, multi-well HF operations were in progress in two horizontal wells (figure 4*a*), wells A and B, both drilled into the Lower Middle Montney zone. Observations of pore pressure in these two horizontal wells (figure 4*b*) indicate that they straddle a significant pressure boundary marked by a difference of approximately 10 MPa over a distance <1.2 km. Since the wells are drilled at slightly more than 2 km depth, this pressure difference equates to 4.5 kPa/m when expressed as a lateral pressure gradient, or 3.74 kPa/m/km when expressed as a horizontal gradient. This large pressure contrast between two closely spaced wells motivates further investigation of a potential association between horizontal gradient in pore pressure and induced seismicity risk.

The 30 November 2018 induced seismicity sequence is characterized by remarkably diverse source mechanisms (figure 4*a*), likely due to the complex structural setting and a stress regime that is transitional from strike-slip to reverse faulting (Salvage & Eaton 2022). The reverse-sense mainshock is interpreted to have reactivated a north-dipping normal fault ([39]; Salvage & Eaton, 2022) that forms the southern bounding fault (SBF) of the Fort St. John Graben, situated within a domain of relatively high pore pressure as shown in figure 2*a*. For the present study, of particular note are several small strike-slip events located proximal to well G that lie approximately on an east-west strike direction (figure 4*a*). These small strike-slip events include a 1.8 M_L_ foreshock on 29 November 2018 (Salvage & Eaton, 2022). It is important to consider that, although precise relative locations were obtained by Salvage & Eaton (2022), there are still relatively large uncertainties (100 sm) in the absolute locations of these events. Therefore, although the strike-slip events appear to be proximal to well G, there is considerable uncertainty with respect to the separation distance between well G and the inferred fault. Despite this, there is high confidence in the orientation of the fault (east-west), the type of faulting (strike-slip) and the positioning of the fault between two wells that exhibit an exceptionally large difference in measured pore pressure.

In summary, this case study shows that within an area of western Canada characterized by elevated levels of fluid-induced seismicity and unusually dense sampling of pore pressure, an apparent spatial association exists between high horizontal gradient of depth-normalized pore pressure and epicentral locations of HF-induced events. Further, in the case of a seismicity sequence associated with the largest induced event (4.5 M_L_), it is notable that fault activation occurred in proximity to two horizontal wells, between which there is an exceptional contrast in pore pressure (~10 MPa). In addition to the location uncertainty noted above, several other sources of uncertainty bear mentioning. First, the large difference between the measured horizontal gradient of 3.75 kPa/m/km between wells G and H and the distribution of horizontal gradient values extracted from the smooth grid (mean of ~0.25 kPa/m/km, figure 3) is most likely an artefact of the smoothing procedure necessitated by the irregular and sparse (in areas) distribution of sample points. Although the spatial trends in figure 2*b* remain significant, smoothing undoubtedly removes short-wavelength features such as the contrast between wells G and H that are more indicative of the actual contrast in pore pressure across sealing faults. Furthermore, because the epicentral locations of seismic events are subject to uncertainty, so too are the extracted values of horizontal gradient. Nevertheless, bootstrap resampling analysis indicates that it is highly unlikely that the apparent high horizontal gradient at epicentral locations occurred purely by chance.

## 3. Numerical simulations

In this section, general geometrical characteristics adapted from figure 4 are used to construct a set of representative numerical simulations that consider a steady-state pore-pressure regime near a sealing fault, the expected pore-pressure distribution at the time of intersection of a vertical strike-slip fault by a hydraulic fracture and possible fault rupture behaviour consistent with a 1.8 M_L_ induced event. For the rupture model, consideration of rate-and-state fault frictional rheology is warranted based on observations from this sequence of hybrid waveform characteristics that mark a transition between aseismic and seismic slip [40]. We remark that although this forward-modelling exercise is informed by data from well logs, HF operations and the induced seismicity sequence, it is intended only to be illustrative of the general characteristics of sealing fault activation.

### (a) Methods

In Cartesian coordinates *x_i_*, *i* = 1, 2, 3, for a poroelastic medium, the pore pressure (*p*) is governed by the diffusion equation [15]


(3.1) $$\frac{\partial }{\partial x_{i}}\left[D_{ij}\frac{\partial }{\partial x_{j}}p\right]=\frac{\partial p}{\partial t},$$

where *D_ij_* are components of the tensor of the hydraulic diffusivity and Einstein’s summation convention is used. For the calculations described below, an isotropic diffusivity tensor is used, leading to


(3.2) $$D_{ij}=D\left[\begin{array}{ccc} 1 & 0 & 0\\ 0 & 1 & 0\\ 0 & 0 & 1 \end{array}\right],$$

where *D* is the isotropic diffusivity scalar (see the electronic supplementary material). Under steady-state conditions, for an isotropic medium, equation (3.1) reduces to


(3.3) $$\frac{\partial }{\partial x_{i}}\left[D\frac{\partial }{\partial x_{j}}p\right]=0,$$

and for a homogeneous material this reduces further to Laplace’s equation


(3.4) $$D\nabla^{2}p\left(t,\text{x}\right)=0.$$

Any linear gradient in *p*, or indeed a constant value of *p*, trivially satisfies equation (3.4) and so can be added to any solution to fit the prescribed boundary conditions. For simplicity, we therefore confine our attention to seeking a solution to equation (3.3) with respect to excess pore pressure, defined as the difference between the true pore pressure and a background pore pressure state expressed using a simple linear depth gradient.

**Figure 5.**
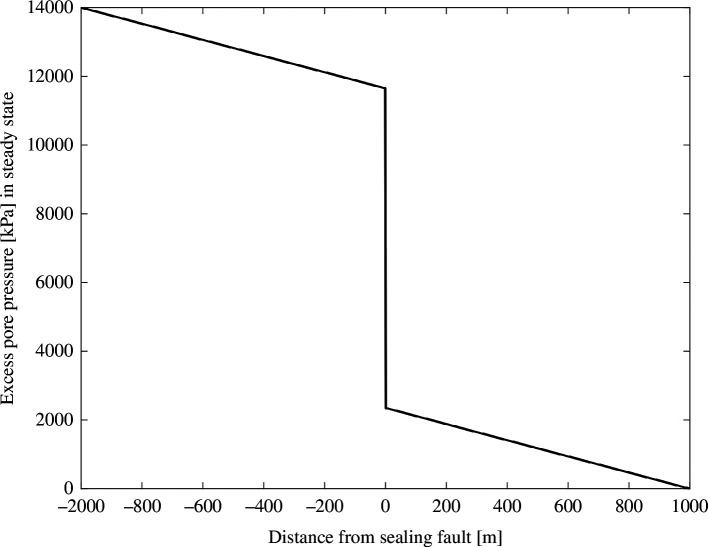
Steady-state excess pore pressure profile across a sealing fault, obtained as a one-dimensional solution to equation (3.1) subject to assumed boundary conditions at [−1000,1000] m. Model parameters are listed in table 1.

Figure 5 shows a one-dimensional solution to equation (3.3) for a model of a sealing fault with parameters listed in table 1, where fixed p boundary conditions are applied at *x* = –1000 m and *x* = 1000 m. The model parameters used here are from Shapiro *et al*. [15], adjusted to fit the conditions of the Montney Formation. In steady state, pore pressure behaves in the same manner as temperature described by the heat equation. Thus, similarly to a steady-state geothermal gradient, we see a linear gradient in *p* with a slope that depends on diffusivity (*D*). As diffusivity tends to zero (i.e. an impermeable fault, much like an ideal thermal insulator in the case of the heat equation), a sharper contrast emerges across the sealing fault.

**Table 1.** Poroelastic model parameters for the steady-state pore-pressure model^a^.

input parameter	value
porosity	10%
background permeability	50 µD
dry frame bulk modulus, *K*_d_	49 GPa
grain material bulk modulus, *K*_g_	75 GPa
fluid bulk modulus, *K_f_*	2.2 GPa
pore fluid dynamic viscosity	1.9 × 10^–4^ Pa^–s^
dry frame shear modulus	22.5 GPa
high-P boundary condition	14 MPa
low-P boundary condition	0 MPa

^a^ Parameters are derived from Shapiro *et al*. [15], modified for the conditions of the Montney Formation.

**Figure 6.**
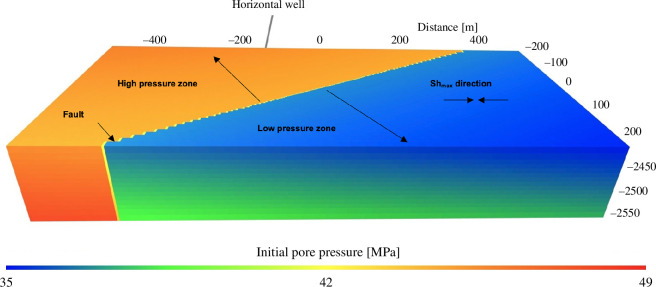
Model set-up for hydraulic fracture simulation and fault-slip simulation.

The one-dimensional solution shown in figure 5 was used to construct a three-dimensional model, depicted in figure 6, by adding a linear depth gradient in *p*. This three-dimensional model was used in numerical simulations for both hydraulic fracture propagation and fault rupture. For the fracture simulation, high-pressure fluid is injected at a point in the model, notionally located along a horizontal well, within a layer characterized by the generalized properties of the Middle Lower Montney zone. The injection point is 150 m distant from a vertical fault surface. This simulation was performed to model hydraulic fracture propagation processes and to estimate fluid pressure at the intersection of the hydraulic fracture and the low-permeability (and low *D*) fault core. ResFrac, a commercial HF and reservoir simulator code, was used. Details of the simulator conceptual model and numerical approach are described by McClure *et al*. [41]. ResFrac is a fully coupled hydraulic-fracturing, reservoir and wellbore simulator that rigorously models the relevant physical processes involved in HF using a finite-volume method for fluid flow and poroelastic stress changes. The simulator is used for both fixed grid-block sizes and variable grid-block sizes. In this study, the matrix element size was refined towards the fault to reduce numerical artefacts.

The model includes three geological units: overburden, target reservoir and underburden. The initial minimum horizontal stress in the overburden and underburden units was set to 100 MPa to ensure that simulated hydraulic fractures do not propagate into the upper and lower geological units. Except for the initial pore pressure, which varies by distance from the fault core as shown in figure 5, other input parameters are uniform. Table 2 summarizes the model parameters used for this simulation, which are modified from Zeinabady *et al*. [42] to ensure consistency with the steady-state model parameters used here. The simulation is isothermal, with a uniform temperature of 82°C set throughout the model. In this simulation case, a total of 1080 m³ of slickwater and 5200 kg of proppant (55 mesh) were injected over a 3-h injection period. A discrete fracture propagation algorithm was used, and fracture toughness was allowed to scale with fracture size (see [41] for more details). Initial vertical and horizontal toughness were set to 3.3 MPa m^1/2^ and relative fracture toughness per square root of fracture size was set to 1 m^−1/2^.

**Table 2.** Model parameters for hydraulic-fracture simulation using ResFrac [41].

input parameter	value	input parameter	value
background permeability, mD	0.05	Young’s modulus, MPa	40 000
fault core permeability, mD	1.25 × 10^−5^	Poisson’s ratio	0.25
porosity, fraction	0.1	reservoir fluid viscosity^b^, cp	0.028
pore pressure at fault core^a^, MPa	42	measured depth, m	5200
minimum horizontal stress, MPa	58	true vertical depth, m	2500
maximum horizontal stress, MPa	90	well inner diameter, m	0.127
fault orientation, degree	31	injection rate, m^3^/min	6
reservoir thickness, m	100	injection duration, h	3

^a^ Pore pressure changes by distance from the fault core (see figure 5). The pore-pressure at the fault core was selected based on the initial measured pore-pressure value within the closest hydraulically fractured well.

^b^ Reservoir fluid viscosity at reservoir temperature (82°C). The simulation is isothermal, and uniform temperature is set throughout the model.

The extended finite element method (X-FEM) proposed by Hosseini *et al*. [43] was then used to solve the coupled problem of fault slip and deformation of the surrounding porous rock, whose geomechanical properties are summarized in table 2. The X-FEM approach is based on a two-dimensional model that also considers out-of-plane strike-slip displacement. The simulation is therefore suitable for oblique-slip reactivation (i.e. a combination of fracture modes I and III) along an existing strike-slip fault. The regularized rate-and-state friction model [44,45] is used to compute the friction coefficient of the fault as a function of slip rate (*V*) and state variable (θ), given by


(3.5) $$\mu \left(V,\theta \right)=\mu_{0}+aln\left(\frac{V+V_{0}}{V_{0}}\right)+bln\left(\frac{\theta V_{0}}{D_{c}}\right);\frac{d\theta }{dt}=1-\frac{\left(V+V_{0}\right)\theta }{D_{c}},$$

where *μ*_0_ is the stationary (static) friction coefficient; *a* and *b* are the direct-effect and friction evolution parameters, respectively, and *V*_0_ is the reference slip rate (creep rate). The state variable, θ, is an indicator of the sliding history, and the characteristic slip length, *D_c_*, is the length over which friction evolves after the fault is perturbed. The evolution of the state variable is determined based on an ageing law. The frictional parameters of the fault were chosen as *μ*_0_ = 0.6, *a* = 0.005, *b* = 0.008, *D_c_* = 100 μm, *V*_0_ = 10^–9^ m/s. These parameters were selected based on published laboratory experiments for similar rock types [46,47].

### (b) Results

**Figure 7.**
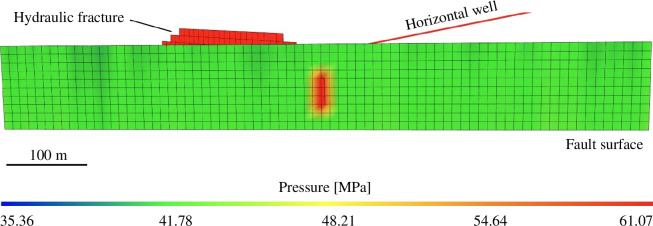
Modelled fluid (pore) pressure within the hydraulic fracture and along the fault surface at the moment when the hydraulic fracture intersects the fault.

As the hydraulic fracture tip approaches the fault core, the fault pore pressure increases from 42.0 to 60.6 MPa. Figure 7 shows the calculated pore-pressure distribution along the fault surface, corresponding to the time at which the hydraulic fracture intersects the fault. As expected, the pressure peaks within the hydraulic fracture. This computed pressure distribution was used as the initial condition for the next step, earthquake nucleation and rupture simulation, as fault slip was not modelled in ResFrac simulation. The pressure-driven hydraulic fracture reaches a vertical strike-slip fault, which is aligned at an angle of 30° with respect to the maximum far-field stress (Salvage & Eaton, 2022). The initial normal effective stress and shear stress on the fault plane are 24 and 13.8 MPa, respectively, which leads to a stress ratio of 0.58 (slightly smaller than the static friction coefficient of the fault) such that the fault is initially close to a critically stressed state. The sudden increase in pore pressure caused by the intersecting HF leads to a reduction in the effective stress on the fault. This causes a subsequent reduction in the shear strength of the fault, such that slip will initiate on the fault.

**Figure 8.**
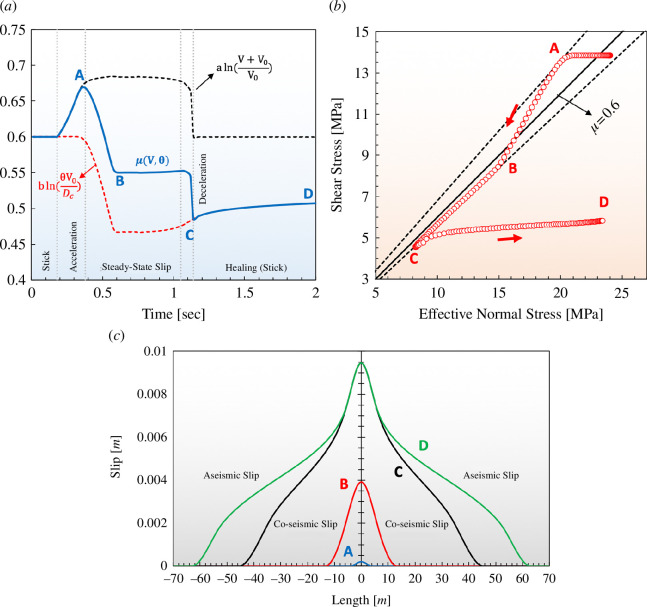
Rupture simulation for a small earthquake on a rate-and-state fault, after initiation by a sudden increase in pore pressure, as depicted in figure 7. (*a*) Graphs showing evolution of friction coefficient (*μ*) and rate-and-state parameters a ln[(V + V_0_)/V0] and b ln[θV_0_/*D*_*c*_] at the rupture nucleation point. Points A–D are described in the text. (*b*) Shear stress versus effective normal stress. (*c*) Snapshots of fault slip distribution versus distance along the fault at times A–D.

Figure 8*a*,*b* shows the variation of friction coefficient, shear and effective normal stresses at the intersection point of the hydraulic fracture and fault. The co-seismic slip at this point undergoes five stages, as follows: (i) *Stick*: the available shear stress is less than the shear strength, and there is no relative movement between fault faces (*V* = 0); (ii) *Slip acceleration*: slip begins and the rate increases so that the rate-dependent term of the friction coefficient is dominant; (iii) *Steady-state slip*: fault slips at a constant rate as the friction coefficient approaches a new steady-state magnitude; since *a – b* < 0, the new steady-state friction coefficient is less than the initial friction (*μ*_0_), leading to a considerable drop in shear stress; (iv) *Slip deceleration*: the slip rate drops suddenly, which causes an abrupt drop in the friction coefficient due to the negative direct effect; and (v) *Healing*: the fault undergoes a sticking stage, at which the friction coefficient takes ~*D_c_*/*V*_0_ to restore its initial stationary friction coefficient. In this stage, the state-dependent part of the friction is dominant.

Figure 8*c* shows the distribution of slip along the fault at different stages of co-seismic slip. The fault undergoes a maximum co-seismic slip of ~1 cm over a distance of ~90 m. Although the regions around the intersection point remain in stick mode, the fault continues to slip aseismically from the rupture tips because the available average shear strength of the fault is less than the far-field shear stress acting on the fault. This aseismic slip stops when all points of the fault are restored to their initial strength.

## 4. Discussion

While the above numerical simulations employ realistic model parameters tailored for this setting, they are intended to be broadly representative of a simple conceptual model of fault-bounded pore-pressure domains. As such, the simulations are meant to be illustrative of the general behaviour of a sealing fault in response to an intersecting hydraulic fracture, rather than necessarily to fit the field observations *per se*. Nevertheless, it is interesting to note that the choice of frictional parameters for the rate-and-state slip simulation, based on independent laboratory experiments for similar rock types, generated a computed slip model for arrested rupture that is consistent, in terms of scale, with a 1.8 M_L_ foreshock observed near well G (figure 4*a*). From figures 7 and 8, the width (*w*) and length (*L*) of the simulated co-seismic slip region are approximately *w* ~ 50 m and *L* ~ 90 m, respectively, and the calculated average stress drop (Δσ) within this region is approximately 4 MPa (see the electronic supplementary material). Using a simple theoretical relationship for stress drop and fault dimensions for a strike-slip fault [48], the seismic moment (*M*_0_) can be expressed as


(5.1) $$M_{0}=\frac{\pi }{2}\Delta \sigma w^{2}L.$$

This leads to a computed moment of *M*_0_ = 1.41 × 10^12^ N m, equivalent to a moment magnitude of *M*_W_ ~ 2, which is in reasonable agreement with the observed foreshock magnitude.

Yu *et al*. [40] analysed the same induced seismicity sequence as the one considered in this study (figure 4*a*) and documented induced events with hybrid-frequency waveforms that are characterized by unusually low spectral corner frequency. Noting the similarity of these hybrid-frequency events with low-frequency earthquakes from plate-boundary fault transition zones, Yu *et al*. [40] argued that this provides evidence for occurrence of aseismic (slow) fault slip during this induced sequence. In the numerical simulation presented here, decelerating slip rate at the end of the rupture process results in a period of slow slip, over a time span of several seconds between times ‘C’ and ‘D’ (figure 8). We speculate that this terminal slow slip phase in our simulation would radiate low-frequency events, which could explain the hybrid-frequency events observed by Yu *et al*. [40].

Within the KSMMA region in our study area, outlined in figures 1 and 2, there is a regulatory requirement for seismic hazard pre-assessments to be carried out prior to drilling [36]. In general, there is a marked tendency for the distribution of HF-induced earthquakes to exhibit distinct spatial clustering [49–51], as clearly expressed in our study region (figure 2*a*)—despite more widespread distribution of HF wells (figure 1*a*). This apparent spatial concentration of fluid-induced seismicity has prompted investigations aimed at understanding possible factors that control geological susceptibility to induced seismicity, including proximity of fluid injections to crystalline basement [52,53], tectonic strain rate [54], proximity of injections to mapped geological features such as ancient reefs [55] and elevated pore pressure [21,50]. The study area considered here lies within the region investigated by Eaton & Schultz [21], who reported statistical evidence for a correlation between elevated pore pressure and likelihood of induced seismicity. The more granular analysis undertaken in the present study points toward an association with high lateral gradient in pore pressure, rather than strictly high pore pressure. These two models reflect different physical mechanisms; in the case of elevated pore pressure, the effective stress is expected to be relatively low, leading to reduced fault strength, whereas here we argue that a zone of high lateral pore-pressure gradient provides a proxy for the presence of a sealing fault system that bounds pore-pressure domains. The nature of the steady-state pore pressure distribution at a sealing fault (figure 5) means that *in situ* pore pressure at fault is expected to be intermediate between adjacent high and low extreme values. Regardless, if low-pressure domains reflect a background pore pressure level (i.e. one that is close to hydrostatic conditions), this model implies that pore pressure at the fault is nevertheless elevated. For pre-operational seismic hazard assessment, a practical utility of this model is that in areas with sufficient data, the pore pressure can be evaluated prior to stimulation [27] to estimate the lateral gradient.

In general, the pore-pressure gradient and intensity of fluid flux across a fault core are dependent on the fault permeability. As shown here, faults with low permeability can cause pressure to build up to steady-state conditions with higher pressure on one side of the fault; by contrast, more permeable faults enable fluid to pass through, leading to a more symmetrical state of pressure balance with a lower lateral pore-pressure gradient across the fault. Furthermore, in the case of a relatively permeable fault system, any overpressure caused by fluid injection on one side of the fault will dissipate more quickly. Previous fault-rupture simulation models generally feature a balanced and symmetrical pore-pressure distribution across the fault, with a negligible lateral gradient (e.g. [43,56–58]). This line of argument implies that systems with low-permeability faults have a greater potential to evolve to a state of incipient frictional failure, such that rupture can occur due to a smaller perturbation in stress conditions.

Nevertheless, the detailed physical mechanisms that could lead to elevated seismic hazard on sealing faults remain an open question. However, previous physics-based simulations provide some important clues. Chang & Segall [16] performed numerical simulations of various scenarios that included sealing (low permeability) faults within a poroelastic medium, subjected to time-varying injection cycles. In the case of sealing faults, their simulations show localized large lateral pore-pressure gradient similar to our steady-state model (figure 5), resulting in equivalent body forces that parallel the fault. Although they demonstrated that direct pore pressure increases for isolated sealing faults are limited, the stress conditions in the surrounding rockmass can create pore-pressure gradients that generate poroelastic stresses that, in some cases, can promote slip [16]. Building on this work, Chang *et al*. [26] considered post-shut-in induced seismicity behaviour and found that sealing faults can exhibit higher seismicity rates than more permeable faults, owing to the combined effects of delayed diffusion and poroelastic stressing.

Reservoir integrity is a key element in the success of CO_2_ storage [59] or underground gas storage (UGS) in depleted hydrocarbon reservoirs. In cases where sealing faults comprise part of the trapping mechanism, as in the Castor UGS project [60,61], the physical model developed here is also applicable. Thus, we believe that it is important to apply findings such as these, developed in data-rich areas of hydrocarbon resource development, to other applications—especially in the context of the global energy transition.

## 5. Conclusion

This study examines sealing (nearly impermeable) faults, with respect to their influence on fluid-induced seismicity. Although they commonly occur as part of structural hydrocarbon traps and may be significant for some CO_2_ or other gas storage projects within depleted hydrocarbon reservoirs, sealing faults have heretofore received scant attention in the induced seismicity literature. A case study from a data-rich unconventional hydrocarbon fairway in northeast British Columbia, Canada, demonstrates that seismicity induced by HF occurs preferentially in areas of high lateral gradient in pore pressure. This association is consistent with a simple one-dimensional steady-state pore-pressure model with an impermeable fault, which shows that a sealing fault is marked by a high lateral pore pressure gradient; it also supports an interpretation that regional high pore-pressure domains are fault-bounded [27].

Examination of pressure data from two horizontal wells that were undergoing HF near the hypocentre of the 30 November 2018 4.5 M_L_ induced earthquake sequence provides direct evidence for an anomalously large (3.75 kPa/m/km) lateral pressure gradient within the unconventional reservoir. A small (1.8 M_L_) foreshock, together with several small aftershocks, appears to delineate a strike-slip fault between the two wells. A set of representative numerical simulations, which combine the conceptual model of fault-bounded pressure domains with realistic model parameters for this setting, shows that a small earthquake could be triggered by the pressure perturbation from an intersecting hydraulic fracture. The numerical simulation exhibits a mix of aseismic and seismic fault slip, consistent with hybrid waveforms observed during this sequence [40]. Taken together our results indicate that sealing faults, which can be identified prior to stimulation using pressure observations, could potentially present an increased risk of fluid-induced seismicity.

## Data Availability

The seismicity data used in this study are available from the BC Energy Regulator seismicity catalogue for events greater than 1.5 M_L_: https://www.bc-er.ca/files/operations-documentation/Induced-Seismicity-Data-and-Submission/main_origin_mag_1point5ml_up.csv. The pore-pressure data illustrated in Figure 4*b* is available through the BC Energy Regulator website in file PST_DTL.csv, available in https://iris.bcogc.ca/download/drill_csv.zip. The applicable wells are G05-22-081-18 and H05-22-081-18. The bulk of the pore-pressure data used in this study were compiled by Enlighten Geoscience [27] and are available at https://www.bcogris.ca/files/projects/pre/er-seismic-2020-01-final-report-enlighten-ver-3.pdf. Additional pore pressure data were compiled by Esmaeilzadeh [30] and are available online [62]. The seismicity catalogue used to create Figure 4 is from [29] and is available in https://gsw.silverchair-cdn.com/gsw/Content_public/Journal/bssa/112/3/10.1785_0120210210/2/bssa-2021210_supplement_table.csv.zip. Supplementary material is available online [63].
